# Fetal Alcohol Spectrum Disorder: What does Public Awareness Tell Us about Prevention Programming?

**DOI:** 10.3390/ijerph16214229

**Published:** 2019-10-31

**Authors:** Peter Choate, Dorothy Badry, Bruce MacLaurin, Kehinde Ariyo, Dorsa Sobhani

**Affiliations:** 1 Social Work, Mount Royal University, Calgary, AB T3E 6K6, Canada; 2 Faculty of Social Work, University of Calgary, Calgary, AB T2N 1N4, Canada; badry@ucalgary.ca (D.B.); bmaclaur@ucalgary.ca (B.M.); kehinde.ariyo1@ucalgary.ca (K.A.); 3 Faculty of Social Work, University of Toronto, Toronto, ON M5S 1A1, Canada; dorsa.sobhani@gmail.com

**Keywords:** Fetal Alcohol Spectrum Disorder (FASD), FASD prevention, FASD awareness, FASD prevention messaging, secondary data analysis

## Abstract

The prevalence of Fetal Alcohol Spectrum Disorder (FASD) does not appear to be diminishing over time. Indeed, recent data suggests that the disorder may be more prevalent than previously thought. A variety of public education programs developed over the last 20 years have promoted alcohol abstention during pregnancy, yet FASD remains a serious public health concern. This paper reports on a secondary data analysis of public awareness in one Canadian province looking at possible creative pathways to consider for future prevention efforts. The data indicates that the focus on women of childbearing age continues to make sense. The data also suggests that targeting formal (health care providers for examples) and informal support (partner, spouse, family, and friends) might also be valuable. They are seen as sources of encouragement, so ensuring they understand the risks, as well as effective ways to encourage abstinence or harm reduction, may be beneficial for both the woman and the pregnancy. Educating people who might support a woman in pregnancy may be as important as programs targeted towards women who may become or are pregnant. The data also suggests that there is already a significant level of awareness of FASD, thus highlighting the need to explore the effectiveness and value of current prevention approaches.

## 1. Introduction

Fetal Alcohol Spectrum Disorder (FASD) results from alcohol use during pregnancy creating lifelong functional and cognitive impairments that fall across a spectrum [1]. The actual prevalence of FASD is unknown, but recent estimates suggest a rate of 1.4%–4.4% of the population in Alberta [2] with 4% identified as the best estimate in Canada [3]. It is thought that the vast majority of people affected are not diagnosed with the disorder either because their health problems are diagnosed under other categories or the individual does not come to the attention of diagnosticians [4]. In essence, FASD often goes unrecognized.

Prevention efforts have been ongoing for over two decades with a focus on broad messaging about the importance of avoiding alcohol use during pregnancy, but these efforts, while creating awareness, have not necessarily changed alcohol use behavior [5,6]. Despite prevention and education efforts, the rates of FASD are thought to be much higher than previously considered, raising concerns that prevention has not been successful in reducing incidence. Hoyme, Kalberg, Elliot et al. [7] indicate that the “soaring prevalence and burden of FASD in children recently led the American Academy of Pediatrics to stress the following: no amount of alcohol intake during pregnancy can be considered safe; there is no safe trimester to drink alcohol; all forms of alcohol pose a similar risk; and binge drinking poses a dose-related risk to the fetus” (p. 2) and, as noted above, many cases of FASD simply go unrecognized by health and helping professionals [4]. We are cautious with this data, as, given that screening and diagnostic criteria and methods are changing, this may confound the prevalence data.

FASD is a clearly established priority for the province of Alberta, Canada [2,8,9], which is where the research reported here was conducted. In 2008, the Government of Alberta implemented an FASD 10-Year Strategic Plan. The first two pillars of that plan focus upon public awareness and education strategies and prevention, while the other pillars look at assessment and diagnosis, support for caregivers, and the presence of an FASD learning organization. This research looks at public awareness, which is part of the first pillar. It uses a secondary data analysis of the 2011 and 2017 datasets to examine characteristics and changes in public awareness of FASD in Alberta. 

The present project looks to understand the degree of awareness of FASD, which can assist in understanding whether the messaging is at least impacting at that level.

## 2. Literature Review

FASD prevention occurs through multiple channels, which include public messaging campaigns, community-based educational programs, outreach, public health programs, direct work with pregnant mothers or those who may become pregnant, criminal justice, and child welfare settings. There are also multiple professions involved, including nursing, social work, midwifery, medicine, and criminal justice [6,10,11]. 

It is not possible to consider FASD messaging as separate from the shame, guilt, and stigma experienced by those diagnosed with the disorder or for the mothers who have delivered a child alcohol-exposed [8,10,11]. The prominent messaging that FASD is a “100% preventable disorder” acts as a foundational message that by just not drinking during pregnancy, the disorder can be prevented, but this does not account for the complexities of women’s lives [12]. Mothers become highly stigmatized in this discourse [13,14]. As Yu, Ahern, Connolly-Ahern and Shen [15] show, the disorder is complex due to its multiple expressions, and the information on prevention is unclear. Connolly-Ahern and Broadway [16] ask the question of why alcohol use in pregnancy is such an emotional topic and indicate that women face competing and confusing narratives such as, no alcohol is best and there is no known safe limit, against other messages that support moderate to light use in pregnancy. Racine, Bell, Zizzo and Green [17] describe messaging as confusing, particularly between the biological aspects of FASD and the messages of stigma and the failure of the mother to self-control. Yu et al. [15] identify message framing as an important aspect that can contribute to prevention, suggesting messages promoting health gains are often effective tools in prevention but discovered “loss frames were more effective in promoting preventative behavior” (p. 698) in relation to FASD. 

Poole et al., [5] have identified four key areas that prevention should focus upon: 1—public awareness and broad health promotion; 2—conversations about alcohol with women of childbearing age and their partners; 3—specialized support for pregnant women, and 4—post-partum support for new mothers [6]. This framework acts as a way to ponder the place of prevention messaging in specific contexts that may allow more focused approaches. These authors have carefully detailed the complexity that exists within each category. Even with this effective framework, the authors note, “significant barriers remain to a coordinated, compassionate, evidence-informed, and systematic approach to prevention tailored to the needs of women at differing levels of risk and their families” (p. 9). Anderson [18], in a review in Ontario, demonstrated that public awareness of FASD was poor. Yu et al. [15] pointed out that college-aged women in their research viewed the possibility that they could have an alcohol-affected pregnancy as a “distant threat” (p. 698). Further, these authors suggest that women’s behavior around alcohol use is the key factor in prevention and suggest that “loss framed messages” (p. 699) appear to be most effective as a tool in prevention. 

The efficacy of messaging needs to be assessed. Doing something that has public appeal is easy to implement but may not assist in changing behaviors. For example, alcohol warning labels have high public acceptance and may impact lower risk consumers but do not seem to be effective for higher risk consumers [19] who may well be trapped in alcohol dependence as well as other social/health problems. Messaging has focused on the women being totally responsible for the safety of the child during the pregnancy, yet the social factors of poverty, violence, addiction, mental health, and historical trauma make assuming that responsibility an overwhelming and often impossible task for the mother [20]. These same authors note that prevention has not been effective in these high-risk populations and that establishing support systems can be problematic. Abusive partners, lifestyles of survival, and intervention styles that only focus on the pregnancy and not the totality of the environmental realities of the woman will tend to fail. Lower risk women may find support systems, such as partners, more readily available, and more open to their own role in prevention.

## 3. Method

### Description of 2011 and 2017 Dataset Methodology

This study is a secondary data analysis of two surveys conducted in 2011 and 2017 by PolicyWise, previously named the Alberta Centre for Child, Family, and Community Research on adult Albertan’s awareness of FASD. Both of these surveys were performed on about 1200 Albertans older than 18 (1205 participants in 2011; 1203 participants in 2017). The samples for both 2011 and 2017 were equally distributed between Metropolitan Edmonton, Metropolitan Calgary, and the remaining areas in Alberta. An equal number of males and females were selected. The 2011 survey used direct dialing, which connected to landlines, whereas the 2017 survey utilized a computerized method, Random Digit Dialing (RDD), to ensure an equal chance of participation. This method of sampling targeted people who had access to landline and cellular phones. Both studies asked a range of questions that studied people’s awareness of FASD in the province. There is a difference in the data collection methodology between the two samples, which limits some of the comparative possibilities. The response rate for 2011 was 26.1%, and 20.4% for 2017. Ethics approval had been granted to the original researchers, which included secondary analysis of anonymized data.

#### Secondary Analysis

This study conducted a secondary analysis of the 2011 and 2017 FASD datasets using SPSS v. 24 (IBM Canada, Toronto, Canada) to examine how awareness of FASD and attitudes towards responsibility as bystanders related to participants’ characteristics (gender, age group, and location in the province) and differed by reporting year. These differences, including between-group comparisons, were examined through descriptive and bivariate comparisons using chi-square tests. 

## 4. Sample Description

The surveys for 2011 and 2017 sampled for a similar proportion of males and females as well as for the region of residence, with a third of the sample each living in Calgary region, Edmonton region, or other parts of Alberta (See Table 1). The sample was more heavily weighted to those 45 years or older, with 60.8% of respondents being 45 years of age or older in 2011 and 71.5% in 2017. Approximately 64% of respondents had no children living in the home in 2011, compared to 71% in 2017. Respondents were primarily Caucasian in 2011 (87.3%), but this decreased slightly in 2017 (81.6%). The majority of all participants were born in Canada in 2011 (81.4%) and 2017 (80.0%). In 2011, 63.1% of respondents identified as being Christian, compared to other religions (6.1%) and no religion (24.9%). In 2017, this shifted to a higher percentage identifying as belonging to another religion (20.5%) and a decrease in the percentage identifying as Christian (47.6%). Our analysis of the increased reporting of religious involvement between the two data sets did not indicate a shift in the prevalence in any specific religious grouping. Approximately 60% of the sample were married in both 2011 and 2017. The demographics suggested that survey respondents were an economically advantaged population for both years of data collection. Approximately 3% identified as making less than $20,000 per year, while more than half made between $60,000 and $124,999 or greater than $125,000 per year. In 2011, 26.3% of respondents did not disclose their income level compared to 19.6% in 2017. This may suggest that hard to reach populations with no access to phones were not reached. In both 2011 and 2017, the majority of the respondents were in relationships (69.0% in 2011 and 67% in 2017), whereas the 2016 Census shows 60.3% of Albertans in relationships (married or common law). This was also a more educated population than the average for the Alberta population [21].

## 5. Results

The data was encouraging in terms of awareness of FASD. In 2011, only 18% of males and 10% of females did not know about FASD. In 2017, awareness grew in that only 15.1% of males, and 5.5% of females were unaware of FASD.

When looking at age categories, in 2017, the 35–44-year age group had the least awareness, but, even there, only 15.6% lacked awareness of FASD. In 2011 the 18–24-year-old age group had 30.3% not aware of FASD. That age group in 2017 is more aware, with only 14.8% lacking awareness. 

Most participants in the 2011 survey agreed that women’s support systems, including her spouse, family, and friends, should assume encouraging roles in supporting her to refrain from alcohol use during pregnancy. There was no significant difference for these support systems by sex, as measured by Chi-square (See Table 2). There were significant differences noted for males and females for external sources of encouragement to not drink during pregnancy; however, a higher percentage of females than males were in favor of healthcare providers (88.9%), the community (78.6%), and the government (73.9%) playing a role. Age of respondent is a significant factor in the decisions about who should be involved in encouraging a woman not to drink alcohol. A higher percentage of respondents under 45 years of age supported all categories compared to respondents who were 45 or older, and all were significant as measured by Chi-square, with the exception of the woman’s family (See Table 3). 

The survey for 2017 shifted the inquiry from “Who should be involved in encouraging a woman…” to “Who should be responsible to support a woman…” and this revision resulted in lower response for the role of personal support systems and external systems. The construct of “responsibility” is unclear as the research question did not clarify what this responsibility would entail. 

In 2017, 29.2% of male participants and 36.4% of female participants indicated that the government was responsible for supporting a woman to not drink during pregnancy, a significant difference noted by Chi-square (See Table 4). A higher percentage of females believed that the community was responsible compared to males. Differences for all other categories were not significant. There were no significant differences noted for responsibility to support the woman by age group (See Table 5).

Respondents were asked about their level of direct knowledge of FASD. In 2017, 48% of participants knew someone that might have FASD, which has increased in comparison to the 34.7% participants in 2011 (See Table 6). As well, in 2017, 28% of participants reported that they knew someone who provided care for someone with FASD, a serious decrease from the 34.1% who reported this in 2011.

## 6. Discussion 

Looking at the data sets from 2011 and 2017, it appears that the awareness of FASD in the province has increased; however, data noted earlier suggests the prevalence is not decreasing [22,23,24]. The current study may indicate that in a general survey, Albertans would show significant awareness of FASD. 

In terms of changes to prevention messaging, the data indicates the focus on women of childbearing age continues to make sense [6]. The data also suggests that targeting formal (health care providers for examples) and informal support (partner, spouse, family, and friends) might also be valuable. They are seen as sources of encouragement, so ensuring they understand risks, as well as effective ways to encourage abstinence or harm reduction, may be beneficial for both the woman and the pregnancy. These supports can be good sources of information as well as validating steps the mother may be taking or considering. The 2017 data continues to support the role of informal support but in more of the role of responsibility. There may well be ethical concerns about shifting responsibility to these parties, but the data continues to suggest the importance of these supports. 

The data also indicates that formal support is an important source of support and knowledge. Choate and Badry [10] found that stigma is a major concern in how women experience interactions with professionals and formal support. Thus, if mothers are able to connect with these supports, that connection is likely to be sustained if the mother is accepted even when struggling with continuing use. Approaches that can flag concerns while still sustaining non-stigmatizing messages may improve the value of formal support in prevention or harm reduction [25,26].

Given the nature of the sample, lower risk populations were mainly surveyed, although they did report having awareness. As is typical of surveys of this nature, the current surveys did not appear to reach higher risk populations with impacts of trauma and social pressures that appear to be prevalent in populations having a child with FASD or a mother using alcohol in pregnancy. Trauma, mental health, socio-economic challenges, domestic violence, and difficulty accessing services belie the individual responsibility model. This raises the pivotal question of whether current prevention approaches have significant efficacy with higher risk mothers.

FASD prevention messages have focused upon abstinence, which attempts to influence the mother’s knowledge and choices. Other factors may need attention, including the contexts in which mothers are making decisions. This might include the mother’s social location and socio-economic status. These elements interact with personal factors such as trauma, addiction, and mental health, which contribute to risk factors related to FASD. Further research is needed in these areas to understand how prevention messaging might directly target such concerns. 

Harm reduction might be one such opportunity. This is worth exploring in more detail as it may shift away from abstinence and stigmatizing messages [10] to ones where traumatized populations may be able to see their lived realities in the messages [27]. This may include acknowledging environmental factors that contribute to the ongoing alcohol use in this population, such as homelessness and lack of social support. A housing-first approach, as well as allocation of funding to this population, may be effective in decreasing or even eliminating alcohol use during pregnancy. 

In these data sets, partners and spouses (which may include those from the LGBTQ+ communities) were seen as an important support for a mother. The role of partners (most often male) is only starting to receive significant consideration [28]. This may indicate a growing opportunity to step away from the constructs of FASD as a primarily female issue [29]. The nature of men’s involvement was not explored further in these surveys. This subject may be controversial because accepting men’s responsibility in regard to pregnant women’s drinking may have implications for the right of women to their bodies. It also assumes that men are available to fulfill this role, which may not be consistent with the lived experiences of traumatized mothers. There is data showing us that paternal alcohol consumption has an impact on maternal health and alcohol consumption [28] This is an area for future consideration [30] as men have not received significant attention in prevention messaging [28]. 

### Limitations

There were some changes in the survey questions between the 2011 and 2017 surveys, which challenge direct comparisons between the two survey points. Some questions used different words, and some were completely modified. For example, in 2011, participants were asked, “Who should be *involved in encouraging* a woman not to drink alcohol during pregnancy?” Whereas, in 2017, participants were asked, “Who is *responsible for supporting* a woman not to drink alcohol during pregnancy”. One may assume that these two questions can bear differential meanings. 

The demographics of the two studies are similar in that the surveys aligned the sample to the representative population for Metropolitan Calgary, Metropolitan Edmonton, and other parts of Alberta. In both data sets, most participants identified themselves as Caucasian, 87.3% in 2011, and 81.6% in 2017. Alberta has a European identity of 70%, 23.5% visible minority, and 6.5% Aboriginal [21]. Thus, the data drew more upon the European descendant population.

A final limitation is that the 2011 survey was conducted by contacting respondents by landlines, which may skew the survey results. The 2017 survey also used mobile phones. Creative survey methods that can directly connect with higher risk populations are needed. This may require more field surveys, the use of outreach programs, connecting in safe injection sites, and street-based health delivery services. There is no doubt these methods are more expensive, harder to successfully complete, and may still fail to connect with the highest risk populations that are disconnected and isolated. Yet, the present work illustrates the challenge of connecting through telephone or similar avenues.

## 7. Conclusions

The results of epidemiological work [21,22,23,24,31] indicate that the true prevalence of FASD may be significantly higher than previous estimates. This work indicates progress with the population at large in this Canadian province using messaging that has been common to FASD prevention programs. The more advantaged populations seem aware of FASD and the need to intervene. 

We remain concerned that effective messaging needs to be further explored with higher risk populations. The present work has not tapped into those populations. This is a significant area for inquiry. We anticipate that trauma-informed understandings and messages are required that connect with a woman’s underlying need for the use of alcohol and other substances [25,26]. 

We also wonder whether prevention messaging is the most effective pathway with higher risk populations. Direct intervention may prove more effective than prevention messaging. We suspect a lack of access to resources may be a stronger or equally strong factor as a lack of knowledge about FASD. Higher risk women may have the knowledge to prevent FASD but have so many needs that are not being met, such as housing and mental health resources and other social determinants of health, that taking steps to prevent alcohol consumption in pregnancy may be too challenging. 

One area that deserves specific attention in the future is the link between trauma in the mother’s life and substance abuse and mental health issues. Proximal stress issues, such as fear of rejection and trauma reminders, can lead to alcohol use [32], while longer-standing trauma increases vulnerability over time with more sustained use [33]. This is a highly vulnerable population and can be very challenging to connect to programming, including participation in research such as the present project. Yet, it would merit focus as Mate [34] indicates that childhood trauma is very prominent in the lives of mothers struggling with substance dependency and living in higher risk situations. They are often exposed to the direct impacts of the trauma along with marginal economic situations, interpersonal violence, sexual assault, and poor access to services. Astely, Bailey, Talbot and Clarren [35] illustrated this point, finding, in a study of 80 mothers who had given birth to a child with FASD, that 95% had experienced physical, sexual, or emotional abuse in their lifetime, 80% had a major mental illness with Post Traumatic Stress Disorder as the most common, and 72% felt unable to reduce their alcohol use as a result of being trapped in an abusive relationship.

The data leads us to believe that the level of awareness of FASD in the general population is reasonable. The data also indicates that respondents view the mother as holding primary responsibility for prevention, although there was reasonable interest in support systems supporting achieving this objective. Even so, the burden is focused upon the mother, which is potentially a significant indicator of where the public attitudes believe prevention should be focused. 

Returning to the work of Poole et al., [5], this work suggests that there is success with the first key area being public awareness and broad health promotion. In our view, though, we seem to need to take that goal and ask about how that helps to address the higher needs populations. This leads us to their second key area, which is conversations with mothers and their partners. If the general population is showing awareness, as the current study suggests, then we can reach higher risk populations and have conversations with them, as Poole et al., [5] contemplate as another key area. 

## Figures and Tables

**Table 1:** Demographic characteristics of participants in 2011 and 2017.

	2011		2017	
	#	%	#	%
**Sex of Respondent**				
Male	600	49.9%	600	49.8%
Female	603	50.1%	605	50.2%
**Age of Respondent**				
18–44	438	36.4%	311	25.8%
45 and Older	732	60.8%	862	71.5%
No Response	33	2.7%	32	2.7%
**Region of Residence**				
Edmonton	401	33.3%	404	33.5%
Calgary	400	33.3%	400	33.2%
Other Alberta	402	33.4%	401	33.3%
**Ethnic Background**				
Caucasian	1050	87.3%	983	81.6%
Non-Caucasian	139	11.6%	199	16.5%
No Response	14	1.2%	23	1.9%
**Children in the Home**				
Children in Home	428	35.6%	346	28.7%
No Children in Home	774	64.3%	853	70.8%
No Response	1	0.1%	6	0.5%
**Religion**				
Christian	759	63.1%	574	47.6%
Other Religion	73	6.1%	247	20.5%
No Religion	300	24.9%	328	27.2%
No Response	71	5.9%	56	4.6%
**Country of Birth**				
Canada	979	81.4%	964	80.0%
Other Than Canada	224	18.6%	239	19.8%
No Response	0	0.0%	2	0.2%
**Marital Status**				
Never Married	181	15.0%	188	15.6%
Married	755	62.8%	733	60.8%
Common-law/Live-in partner	74	6.2%	75	6.2%
Divorced	90	7.5%	100	8.3%
Separated	23	1.9%	32	2.7%
Widowed	77	6.4%	69	5.7%
**Education**				
Less than High school	99	8.2%	80	6.6%
High School Complete	223	18.5%	190	15.8%
Post-secondary	881	73.2%	926	76.8%
No Response	0	0.0%	9	0.7%
**Income**				
Less than 20,000	35	2.9%	41	3.4%
20,000–59,999	197	16.4%	252	20.9%
60,000–124,999	399	33.2%	364	30.2%
More Than 125,000	256	21.3%	312	25.9%
No Response/Don’t know	316	26.3%	236	19.6%
**Total Investigations**	1203	100.0%	1205	100.0%

Percentages are column percentages.

**Table 2:** Who should be involved in encouraging a woman not to drink alcohol during pregnancy by sex of respondent (2011 data).

	Male		Female	
Who Should Be Involved	#	%	#	%
Partner or Spouse ^NS^	532	92.2%	552	93.1%
Woman’s Family ^NS^	542	93.9%	564	95.1%
Woman’s Friend ^NS^	497	86.1%	532	89.7%
Healthcare provider *	488	84.6%	527	88.9%
The Community **	410	71.1%	466	78.6%
The Government **	377	65.3%	438	73.9%
Total Investigations	577	100.0%	593	100.0%

Percentages are column percentages, significance * *p* ≤ 0.05, ** *p* ≤ 0.01, NS—not significant.

**Table 3:** Who should be involved in encouraging a woman not to drink alcohol during pregnancy by age category of respondent (2011 data).

	Under 45		45 & Older	
Who Should Be Involved	#	%	#	%
Partner or Spouse **	405	92.5%	648	88.5%
Woman’s Family ^NS^	405	92.5%	671	91.7%
Woman’s Friend ***	437	99.8%	616	84.2%
Healthcare provider *	380	86.8%	605	82.7%
The Community ***	251	57.3%	502	68.6%
The Government ***	325	74.2%	469	64.1%
Total Investigations	438	100.0%	732	100.0%

Percentages are column percentages, significance * *p* ≤ 0.05, ** *p* ≤ 0.01, *** *p* ≤ 0.001, NS—not significant.

**Table 4:** Who is responsible for supporting a woman not to drink alcohol during pregnancy by sex of respondent (2017 data).

	Male		Female	
Who Should Be Involved	#	%	#	%
Partner or Spouse ^NS^	375	62.5%	386	63.8%
Woman’s Family ^NS^	369	61.5%	386	63.8%
Woman’s Friend ^NS^	351	58.5%	365	60.3%
The Woman Herself ^NS^	408	68.0%	414	68.4%
The Community *	242	40.3%	286	47.1%
The Government **	175	29.2%	220	36.4%
All the above ^NS^	251	41.8%	271	44.8%
**Total Investigations**	**600**	**100.0%**	**605**	**100.0%**

Percentages are column percentages, significance * *p* ≤ 0.05, ** *p* ≤ 0.01, NS—not significant.

**Table 5:** Who is responsible for supporting a woman not to drink alcohol during pregnancy by age category of respondent (2017 data).

	Under 45		45 & Older	
Who Should Be Involved	#	%	#	%
Partner or Spouse ^NS^	208	65.7%	534	62.1%
Woman’s Family ^NS^	208	65.7%	529	61.6%
Woman’s Friend ^NS^	201	62.1%	498	57.9%
The Woman Herself ^NS^	218	68.1%	584	67.8%
The Community ^NS^	148	44.9%	368	42.6%
The Government ^NS^	113	34.5%	278	32.1%
All the above ^NS^	127	42.2%	385	44.3%
**Total Investigations**	**311**	**100.0%**	**862**	**100.0%**

Percentages are column percentages, NS—not significant.

**Table 6:** Comparison of knowledge of Fetal Alcohol Spectrum Disorder (FASD) between 2011 and 2017.

Knowledge of FASD	#	%	#	%
Know anyone you think might have FASD **	418	34.7%	553	48.0%
Know anyone who provided care for FASD **	410	34.1%	340	28.0%
Total Investigations	1203	100.0%	1205	100.0%

Percentages are column percentages, significance ** *p* ≤ 0.01, NS—not significant.
